# Recent advances in endothelial colony-forming cells: from the transcriptomic perspective

**DOI:** 10.1186/s12967-024-05108-8

**Published:** 2024-03-26

**Authors:** Yaqiong Liu, Caomhán J. Lyons, Christine Ayu, Timothy O’Brien

**Affiliations:** Regenerative Medicine Institute (REMEDI), Biomedical Sciences Building, University of Galway, Galway, Ireland

**Keywords:** Endothelial colony forming cells, Transcriptome analysis, Cell dysfunction, Heterogeneity

## Abstract

Endothelial colony-forming cells (ECFCs) are progenitors of endothelial cells with significant proliferative and angiogenic ability. ECFCs are a promising treatment option for various diseases, such as ischemic heart disease and peripheral artery disease. However, some barriers hinder the clinical application of ECFC therapeutics. One of the current obstacles is that ECFCs are dysfunctional due to the underlying disease states. ECFCs exhibit dysfunctional phenotypes in pathologic states, which include but are not limited to the following: premature neonates and pregnancy-related diseases, diabetes mellitus, cancers, haematological system diseases, hypoxia, pulmonary arterial hypertension, coronary artery diseases, and other vascular diseases. Besides, ECFCs are heterogeneous among donors, tissue sources, and within cell subpopulations. Therefore, it is important to elucidate the underlying mechanisms of ECFC dysfunction and characterize their heterogeneity to enable clinical application. In this review, we summarize the current and potential application of transcriptomic analysis in the field of ECFC biology. Transcriptomic analysis is a powerful tool for exploring the key molecules and pathways involved in health and disease and can be used to characterize ECFC heterogeneity.

## Introduction

The term endothelial progenitor cells (EPCs) was first proposed by Asahara et al. in 1997 [1] to describe a subset of CD34 + and VEGFR2 + mononuclear cells, which displayed endothelial characteristics. In this study, these putative EPCs had “spindle-shaped” morphology, expressed endothelial cell markers, took up DiI-labeled acetylated low-density lipoprotein, and incorporated into newly formed vessels in vivo. However, subsequent studies found that these putative EPCs were bone marrow–derived myelomonocytic progenitors rather than true endothelial progenitor cells [2–8]. These putative EPCs did not form new blood vessels themselves but only contributed to the formation of new blood vessels by secreting proangiogenic factors, making it controversial labelling them as EPCs [9–11]. To address this ambiguity, a consensus has suggested to rename these putative EPCs as myeloid angiogenic cells (MACs) [12].

In 2004, Ingram et al. discovered the true EPCs with robust sprouting vascular ability, which are now termed endothelial colony-forming cells (ECFCs) [3]. ECFCs are defined as a rare cell type with strong clonal potential, which can give rise to endothelial cells and generate blood vessels [13]. ECFCs express stem cell markers CD34 and some endothelial markers (such as CD31, VE-cadherin, von Willebrand factor (vWF), VEGFR2) while lacking leukocytic markers (CD45 and CD14) [13]. ECFCs have been successfully isolated from various sources, including cord blood (CB) [14], peripheral blood (PB) [15], fat tissue [16], placenta (PL) [17], and lungs [18]. However, researchers have applied different approaches for isolation and cultivation of ECFCs [see review [19]]. Among these sources, it is invasive to isolate ECFCs from fat and lung tissues. PB is the most accessible, and often the only option for autologous applications [20]. CB is also attractive due to the relatively high frequency [3, 21] and superior angiogenic ability of CB-ECFCs [17], however it suffers from the potential to form an allogeneic immune response post transplantation [22].

**Fig. 1:**
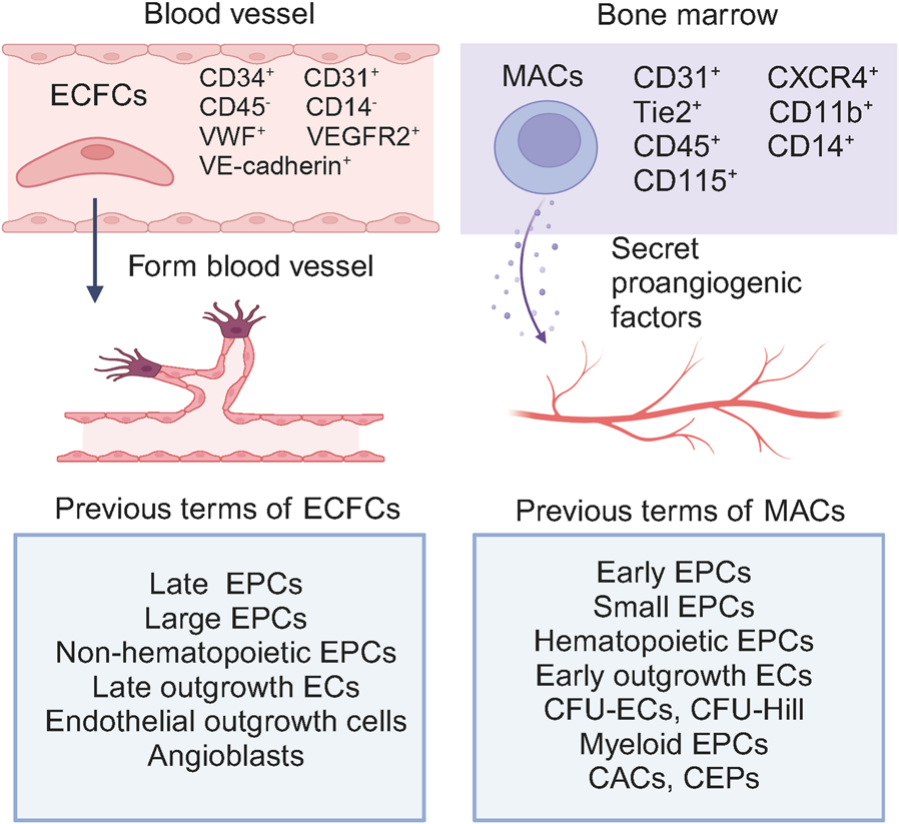
Comparison of endothelial colony-forming cells (ECFCs) and myeloid angiogenic cells (MACs). ECFCs and MACs differ in terms of their source of origin, cell surface marker, and their specific roles in angiogenesis. EPCs: endothelial progenitor cells, ECs: endothelial cells, CFU-ECs: endothelial cell colony-forming units, CFU-Hill: colony forming unit-Hill, CACs: circulating angiogenic cell, CEPs: circulating endothelial progenitor cell

It is worth emphasizing that ECFCs and MACs are two distinct cell types with different cell markers and biological function [23, 24] (Fig. 1). There are several aspects to distinguish ECFCs from MACs: (i) cell markers: ECFCs do not express the leukocytic markers CD45 and CD14 while MACs have high expression level of CD45 and CD14; (ii) angiogenic capacity: ECFCs are able to form stable and functional blood vessels in vivo while MACs cannot generate angiogenic tube-like structures or sprouts; and (iii) phenotype: ECFCs have the ability to differentiate to the endothelial lineage while MACs exhibit a phenotype similar to M2 macrophages [25].

**Fig. 2:**
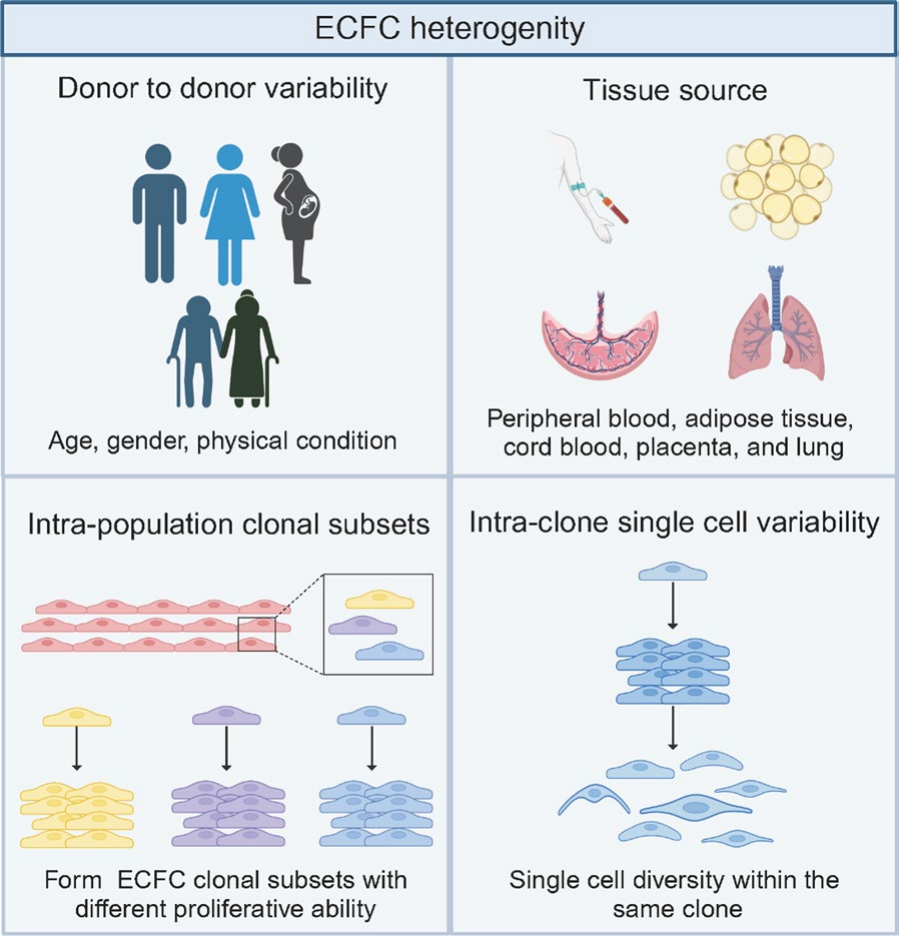
ECFCs heterogeneity. ECFCs exhibit heterogeneity among donors, tissues of origin, clonal subsets, and at the single cell level

Numerous studies have reported that ECFCs exhibit promising vasculogenic ability in xenograft models with ischemic-related disorders [26, 27]. However, as of now, no clinical trials worldwide are being or have been conducted to study the therapeutic effects of ECFCs. To advance ECFCs for clinical development, there are several challenges that need to be addressed. First, the frequency of PB-ECFCs is about 1.7 PB-ECFCs per 1 × 10^8^ peripheral blood mononuclear cells (PBMNCs) [28] and the frequency of CB-ECFCs is approximately 50 CB-ECFCs per 1 × 10^8^ cord blood mononuclear cells (CBMNCs) [29]. The frequency of PB-ECFCs and CB-ECFCs is too low to reach the therapeutic cell dose with limited culture time [30]. Second, the literature has used a diverse array of extracellular matrix coatings and media formulations to isolate and expand ECFCs which impacts the frequency and functionality of ECFCs. Standardised methods for isolating and expanding ECFCs in vitro before transplantation are needed [31]. Third, it is necessary to consider the factors that may lead to the heterogeneity of ECFCs (Fig. 2), such as donor-to-donor variability, tissue origin, clonal subsets, and even single cell from the same clone [32, 33]. Last, it is important to explore genetic or epigenetic modification to improve the dysfunctional ECFCs in disease states [26]. However, the safety and efficacy of genetic or epigenetic modification strategies are in need of further attention.

## Transcriptome analysis

Microarray and bulk RNA sequencing are two commonly used methods to detect gene expression with low cost and high efficiency. The principle of microarray is the pre-designed probe hybridization with target transcripts [34]. Probes will hybridize to complementary transcripts that have been labelled with fluorescent dyes. The fluorescence intensity can be detected with a laser, thus reflecting the relative abundance of transcripts. In bulk RNA sequence, sample RNA is converted to a library of complementary DNA fragments and then sequenced on a high-throughput platform [34]. Gene expression level is measured by the number of reads mapped to a gene. Although microarray and bulk RNA sequencing have achieved great progress in uncovering the genetic mechanism of normal and disease-related physiological processes, researchers could only obtain the average gene expression data for a cell population [35]. Consequently, microarray and bulk RNA sequencing may not provide insight into the important transcriptional signals in individual cells. With the advent of single-cell RNA sequencing, it has rapidly improved the ability to decipher the cell type composition of complex tissues. Limiting dilution, fluorescence activated cell sorting, and microfluidic technology are commonly used techniques to isolate an individual cell [36]. Single cell sequence has been applied to explore the biology of stem cells, including the following aspects: (i) define cell identity and identify rare cell types in complex tissues; (ii) understand lineage progression using Pseudotime [37]; and (iii) identify transcriptional heterogeneity during the process of development or diseases at the single cell level [35].

**Fig. 3:**
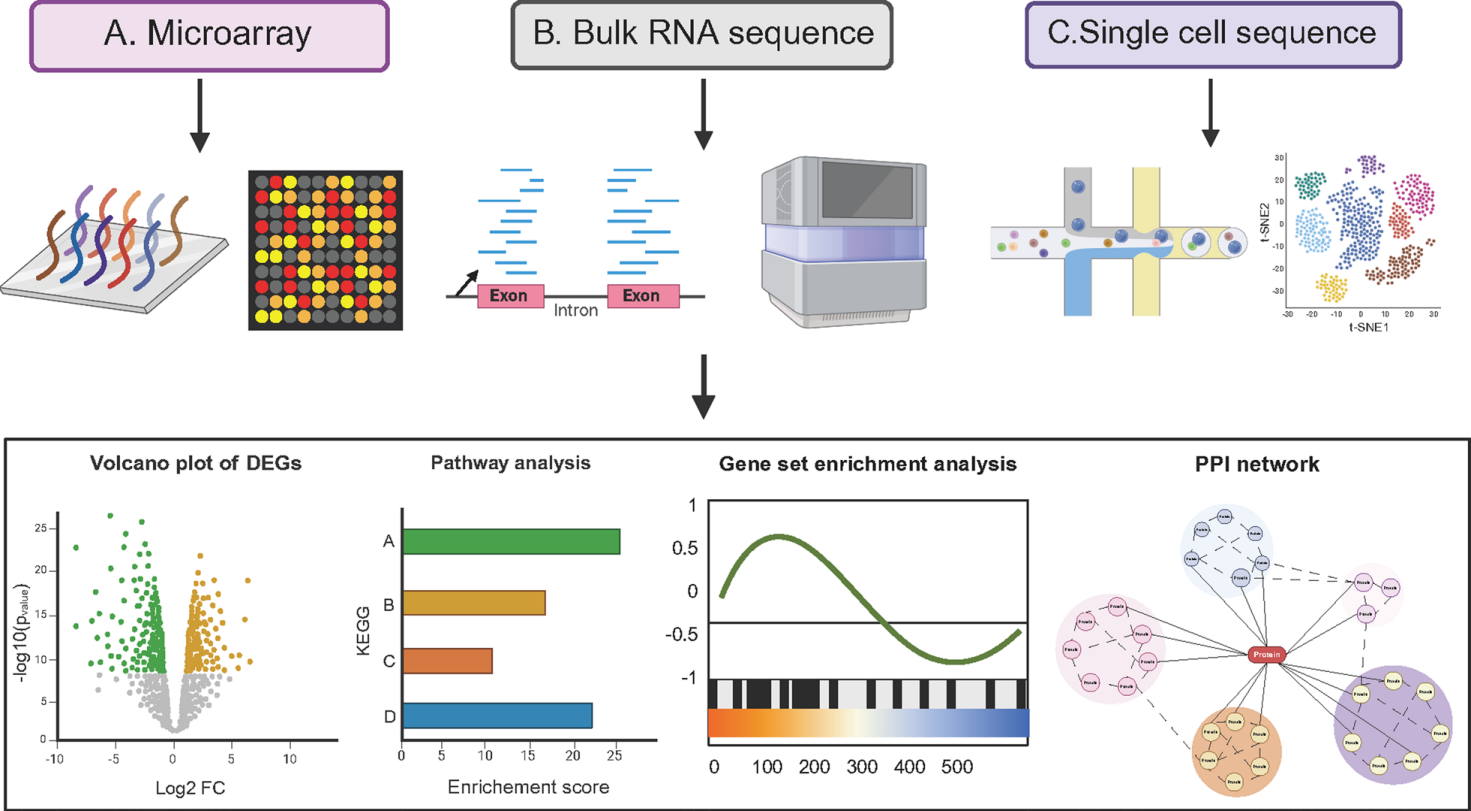
Three transcriptomic analytic approaches: microarray (**A**), bulk RNA sequence (**B**), and single cell sequence (**C**). The three transcriptomic approaches have different working principle to detect gene expression of ECFCs. The bioinformatics analysis is applied to identify key genes and pathways. The DEGs from different groups can be visualized using volcano plot. Kyoto encyclopedia of genes and genomes (KEGG) and gene set enrichment analysis can be performed to identify key pathways. Protein protein interaction (PPI) network is able to show the association of proteins

Microarray, bulk RNA sequencing, and single cell sequencing have been used to explore the key genes and pathways of ECFCs in physiology and pathology (Fig. 3). Transcriptome analysis may provide insight into the origin and characteristics of ECFCs, heterogeneity of ECFCs from different sources, and altered phenotype of ECFCs in healthy and diseased states. One application of transcriptomic analysis is to distinguish closely related cell types. Transcriptomic analysis has some advantages in distinguishing closely related cell types when compared with flow cytometry, immunohistochemistry or polymerase chain reaction [5]. Firstly, transcriptomic analysis can provide the whole gene expression profile instead of only a few cell markers. Secondly, microarray and bulk RNA sequence can allow the clustering algorithm [38] to construct a hierarchy of relatedness that may reflect the taxonomy in biologic hierarchies. Single cell sequence, with its better resolution, is particularly effective in discerning among closely related cell types by measuring the gene expression at the individual cell level [39]. Another application of transcriptomic analysis in ECFCs is detecting the pivotal pathways and mechanisms that are affected in various disease states. These pathways and mechanisms could provide useful information for improving our understanding of ECFC functionality.

In this review, we provide an overview of the published ECFC transcriptome data, which are associated with the origin of ECFCs, the phenotype and functional characteristics of ECFCs, and key modules and pathways of ECFCs under disease states (Table 1). Not only may it facilitate the secondary data mining of publicly available ECFC datasets to generate new hypotheses, but also it will contribute to overcoming the obstacles to the clinical application of ECFCs.

**Table 1:** Overview of published ECFC transcriptome data

Sources	Method	Comparison groups	Available dataset	Main results	Refer-ences
CB-EC-FCs	Single cell sequence	CBMNCs, HUVECs, and CB-ECFC-s	GSE220468	CB-ECFCs may originate from resident vascular endothelial cells, displaying transcriptome features resembling HUVECs more than any other CBMNC clusters	[43]
PB-ECF-Cs	RNA sequence	HCAECs, HUVECs, and PB-ECFC-s	GSE131995	PB-ECFCs express endothelial cell markers of artery, vein, and lymph-vessel, which serve as an intermediate population between HCAECs and HUVECs	[44]
PB-ECF-Cs	Microarray	ECFCs, AT-ECs and HUVECs	GSE55695	GO analysis shows that the differences between PB-ECFCs and AT-ECs are within the areas of glycosaminoglycan and heparin binding, vasculature development, extracellular matrix and cell adhesion. The difference between PB-ECFCs and HUVECs is antigen processing and presentation	[45]
PB-ECF-Cs	RNA sequence	HMECs and PB-ECFC-s	GSE74322 and GSE54416	GO analysis shows enrichment for the terms “cell adhesion” in HMECs and “single-multicellular organism process” in ECFCs	[46]
CB-EC-FCs	Small RNA sequencing	CB-ECFC-s and HUVECs	–	CB-ECFCs have lower levels of two anti-angiogenic microRNAs (miR-221 and miR-222) than HUVECs. miR-221-PIK3R1 and miR-222-ETS1 pairs are deregulated in PB-ECFCs from CAD patients	[47]
PB-ECF-Cs	Single cell sequence	PB-ECFC-s and HUVECs	NCBI dBGaP system: phs002731.v1.p1	PB-ECFCs exhibit a venous phenotype due to the low expression level of artery markers and high expression of venous markers	[48]
PB-ECF-Cs	RNA sequence	PB-ECFC-s and lymphatic ECFCs	GSE54416	Pathway analysis of the top DEGs between PB-ECFCs and lymphatic ECFCs reveals that these DEGs play an important role in regulation of cell differentiation, vasculature development, and endothelial cell differentiation	[46]
CB-EC-FCs	MiRNA microarray	CB-ECFC-s and PB-ECFC-s	–	miR-193a-3p reduces the angiogenic ability of CB-ECFCs and PB-ECFCs by targeting HMGB1	[52]
CB-EC-FCs	Microarray	CB-ECFC-s and PB-ECFC-s	GSM50853-5 GSE20283	Compared with PB-ECFCs, CB-ECFCs have higher expression of osteogenic and angiogenic genes	[53]
CB-EC-FCs	Microarray	CB-ECFC-s and PL-ECFCs	–	CB-ECFCs and PL-ECFCs have similar gene expression profiles	[17]
CB-EC-FCs	MiRNA sequence	Control group and ECFC EVs	–	ECFC EVs deliver miR-21-5p and inhibit THBS1 expression to promote endothelial cell repair	[56]
PB-ECF-Cs	MiRNA sequence	ECFC and ECFC EVs	–	ECFC EVs ameliorate myocardial infarction by shuttling miR-218-5p/miR-363-3p to modulate the p53/JMY signalling pathway	[57]
CB-EC-FCs	MiRNA sequence	CB-ECFC-s exosomes and microparticles	–	The transfer of CB-ECFC-EVs with enriched miR-486-5p to the kidney demonstrates protective effects against ischemic kidney injury	[58]
PB-ECF-Cs	MiRNA sequence	PB-ECFC EVs under normoxic and hypoxic conditions	–	miR-10b-5p is enriched in PB-ECFC EVs under normoxic conditions. PB-ECFC EVs enriched with miR-10b-5p alleviate fibrosis by targeting the fibrotic genes Smurf1 and HDAC4	[59]
PB-ECF-Cs	MiRNA microarray	PB-ECFC EVs	–	ECFC EVs with miR-21-5p regulates autophagic flux to promote vascular endothelial repair by inhibiting SIPL1A2 in atherosclerosis	[60]
CB-EC-FCs	MiRNA sequence	CB-ECFC and ECFC EVs	–	In vivo and in vitro, CB-ECFC EVs promote angiogenesis during ischaemic retinopathy The top five miRNAs enriched in CB-ECFC EVs compared to CB-ECFCs were miR-4532-5p, miR-451a-5p, miR-7704-5p, miR-486–2-5p, and miR-486–1-5p	[61]
CB-EC-FCs	RNA sequence	hypoxia	GSE142123	Hypoxia impairs the initial outgrowth of CB-ECFCs and reduces the proliferation of cultured PB-ECFCs Gene expression profiles of PB-ECFCs under hypoxia show the regulation of the cell cycle and metabolism as major altered gene clusters	[63]
CB-EC-FCs	Microarray	CB-ECFC-s under normoxic oxygen and hypoxic conditions	–	The DEGs of CB-ECFCs under hypoxic conditions are involved in cell apoptosis, cell cycle and MAPK pathways	[64]
CB-EC-FCs	Microarray	CB-ECFC-s under normoxic and hypoxic conditoins	–	The PLAC8–NOX4 signalling axis improves the angiogenic functions of CB-ECFCs exposed to hypoxia	[65]
CB-EC-FCs	RNA sequence	ECFCs under normoxic and hypoxic conditions	GSE142123	ANGPTL14, ENO2, ETXNIP, and SLC2A3 were upregulated while VEGFR2, NOS3, and FLT1 were downregulated. Although the HIF1 pathway is activated, there is no significant enrichment for the VEGFA pathway	[66]
CB-EC-FCs	Microarray	PT-ECFCs and CT-ECFCs	ArrayExpress database: E-MTAB-4860	Biogenesis of pro-senescent microparticles by PT-ECFCs is driven by SIRT1-dependent epigenetic regulation of MKK6	[67]
CB-EC-FCs	RNA sequence	CB-ECFC-s of lean, overweigh-t and GDM pregnancie-s	GSE228990	Higher gestational weight gain delays wound healing and reduces expression of long non-coding RNA KLRK1-AS1 in neonatal endothelial progenitor cells	[70]
CB-ECFCs	Methylation array	ECFCs from healthy women and women with preeclamp-sia	–	DNA methylation of foetal ECFCs is affected in preeclampsia	[75]
CB-EC-FCs	MiRNA sequence	ECFCs from healthy women and women with preeclamp-sia	–	miR-1270 is downregulated in ECFCs from women with preeclampsia. The downregulation of miR-1270 inhibits tube formation capacity and chemotactic motility	[76, 77]
CB-EC-FCs	Microarray	ECFCs from healthy pregnancie-s and GDM pregnancie-s	–	Knockdown of PLAC8 improves proliferation and senescence defects of ECFCs from GDM pregnancies	[78]
PB-ECF-Cs	MiRNA sequence	ECFCs from healthy donors and CAD patients	–	miR-410-3p, miR-497-5p, and miR-2355-5p are upregulated in CAD-ECFCs. Knockdown of these miRNAs can restore the expression of VEGFR2 and increase angiogenic activities of CAD-ECFCs	[82]
PB-ECF-Cs	MiRNA sequence	ECFCs from healthy donors and CAD patients	–	miR-146a-5p and miR-146b-5p are increased in CAD-ECFCs. miR-146a-5p and miR-146b-5p impair angiogenesis ability by targeting RHOJ	[83]
PB-ECF-Cs	Microarray	ECFCs from healthy donors and DM patients	GSE43950	A total of 822 upregulated and 148 downregulated genes are identified as DEGs. IL8 and CXCL1 may lead to the pathophysiology of DM-ECFCs	[86]
PB-ECF-Cs	Microarray	ECFCs from healthy donors and patients with PDR	–	Two anti-angiogenic genes (TSP1 and TIMP-3) are upregulated in PDR-ECFCs	[87]
PB-ECF-Cs	MiRNA sequence	ECFCs from healthy donors and patients with IPAH and HPAH	–	Upregulated miR-124 reduces the expression of glycolysis related genes and proliferative abnormalities of PB-ECFCs from PAH	[89]
PB-ECF-Cs	Microarray	ECFCs from BC and RCC	–	Compared with ECFCs from healthy donors, BC-ECFCs and RCC-ECFCs shared 35 DEGs, 10 of which are organized in a gene network centred on FOS	[93]
PB-ECF-Cs	Microarray	ECFCs from healthy donors and MDS	–	The frequency and cell adhesion ability of ECFCs are increased in MDS patients. MDS-ECFCs show a hypermethylated phenotype and have a lower expression of several Wnt pathway constituents. The addition of soluble Wnt3A could partially rescue the defects of MDS ECFCs	[95]
PB-ECF-Cs	MiRNA sequence	ECFCs from control group and type 1 VWD patients	–	ECFC from control group and type 1 VWD patients show DEGs and miRNAs, which may lead to the pathogenesis of type 1 VWD	[97]
PB-ECF-Cs	Single cell sequence	ECFCs from control group and patients with low VWF levels	NCBI dBGaP system: phs002731.v1.p1	FLI1 is identified as the candidate gene that mediated the expression level of VWF	[48]
PB-ECF-Cs	Microarray	ECFCs from control group and patients with uVTE	GSE118259	The activation of TNFSF15–TNFRSF25 axis reduces survival and proliferation of ECFCs in uVTE patients	[99]
PB-ECF-Cs	Microarray	ECFCs from control group and patients with MMD	–	RALDH2 is decreased in MMD ECFCs due to defective acetyl-histone H3 binding to the promoter region. Knockdown of RALDH2 in normal ECFCs induces decreased capillary formation in vitro. The panobinostat is a potent therapeutic option for MMD patients	[101]
PB-ECF-Cs	Microarray	ECFCs from control group and patients with MMD	–	CDKN2A is upregulated in MMD ECFCs. The knockdown of CDKN2A enhances the cell growth and tubule formation ability of MMD ECFCs	[103]
PB-ECFCs	Microarray	ECFCs from control group and patients with MMD	–	Hypomethylation at the SORT1 promoter CpG sites leads to increased expression of SORT1 in MMD PB-ECFCs. SORT1 overexpression inhibits the tube formation of HUVECs	[104]

## Applications of transcriptome analysis in ECFC research

### The origin of ECFCs

Ongoing debates have occurred spanning two decades regarding the origin of ECFCs, with several studies attempting to determine whether these cells originate from bone marrow or blood vessel wall using various approaches. Lin et al. analysed blood samples from bone marrow transplant recipients who had received gender-mismatched transplants and found that circulating endothelial cells with high proliferative capacity (currently known as ECFCs) possessed donor genotype rather than recipient genotype. This evidence suggested that ECFCs may be not derived from the recipient blood vessel wall, but from the donor bone marrow [40]. Nevertheless, there are a series of studies that presented a different perspective. Yoder et al. investigated the endothelial cell colony-forming units (CFU-ECs) and ECFCs generated from polycythemia vera patients bearing a JAK2 V617F mutation. The hematopoietic-derived cells were expected to display JAK2 mutation. In this case, the CFU-ECs displayed JAK2 mutation while most ECFCs did not harbour the JAK2 mutation. Moreover, CFU-ECs would differentiate into macrophages rather than form perfused vessels. This study provided compelling evidence that ECFCs were not derived from hematopoietic stem cells [28]. Fujisawa et al. also used the sex-mismatch bone-marrow transplantation patients to explore the origin of ECFCs. They proved that ECFCs exhibited recipient genotype rather than donor genotype, which indicated that ECFCs did not originate from the bone marrow [41]. To reconcile these contradictory findings, there is a need to investigate the gene expression profile of cells collected both from blood and vessel wall [42]. Recently, Lin et al. conducted a single cell sequence of CBMNCs, human umbilical vein endothelial cells (HUVECs), and ECFCs. This study has significant implications in clarifying the origin of ECFCs. The results revealed that the gene expression pattern of ECFCs was more similar to HUVECs than CBMNCs clusters. The protein C receptor (PROCR) has emerged as a novel marker of CB-ECFCs. Pseudotime analysis and lineage tracing of mice showed that ECFCs were derived from resident vascular endothelial cells instead of bone marrow. As mentioned above, single cell sequence and lineage tracing provided evidence that ECFCs may be derived from the blood vessel wall [43]. However, it should be noted that ECFCs isolated from mice may not always reflect human ECFCs physiology. In addition to single cell sequence transcriptomic analysis, it may be necessary to conduct a multi-layered single-cell-omics approach to determine the intrinsic properties and spatial context of ECFCs.

### Comparing the characteristics of ECFCs with mature endothelial cells

To characterize ECFCs, the initial transcriptomic studies primarily focused on comparing ECFCs with common endothelial cells from various sources. Kutikhin et al. used bulk RNA sequencing to explore the gene expression profiles of PB-ECFCs, HUVECs, and human coronary artery endothelial cells (HCAECs). The gene expression profiles demonstrated that PB-ECFCs were highly similar to HUVECs and HCAECs. But there was still a slight distinction among the three cell types. The lymphatic endothelial cell marker LYVE1 was highly expressed in PB-ECFCs. Additionally, PB-ECFCs exhibited elevated levels of the venous endothelial marker NRP2 in comparison to HCAECs. At the same time, PB-ECFCs had higher expression of arterial differentiation marker NOTCH4 than HUVECs. Thus, PB-ECFCs could be regarded as a transitional cell type between HUVECs and HCAECs [44]. Krisztina et al. analysed the transcriptome sequencing of PB-ECFCs, HUVECs, and endothelial cells isolated from adipose tissue (AT-EC). Differently expressed genes (DEGs) observed between PB-ECFCs and AT-EC were related to pathways associated with: glycosaminoglycan and heparin binding, vasculature development, extracellular matrix and cell adhesion. Gene ontology (GO) analysis of DEGs between PB-ECFCs and HUVECs uncovered a significant enrichment in antigen processing and presentation [45]. Another study compared the gene expression patterns of ECFCs and human microvascular endothelial cells (HMECs) to explore the features of ECFCs. GO analysis showed enrichment for the terms “cell adhesion” in HMECs and “single-multicellular organism process” in ECFCs [46]. As in vivo studies have shown that the angiogenic ability of HUVECs were not as strong as CB-ECFCs, small RNA sequencing was done to decipher the miRNA expression patterns in HUVECs and CB-ECFCs. Two anti-angiogenic miRNAs, miR-221 and miR-222 had higher expression in HUVECs than CB-ECFCs [47]. Additionally, miR-221 and miR-222 were upregulated in PB-ECFCs from patients with coronary artery disease (CAD). miR-221 and miR-222 acted as negative regulators of angiogenesis by targeting PIK3R1 and ETS1, respectively. Overexpression of miR-221 and miR-222 inhibited cellular motility and tube formation ability of PB-ECFCs in vitro and hindered vasculogenesis in a zebrafish model [47]. A recent single-cell sequencing study reported that venous markers (NRP2, EPHB4) were highly expressed in PB-ECFCs and arterial markers (EFNB2, NOTCH1) were relatively low in PB-ECFCs when compared with HUVECs, indicating that PB-ECFCs were of a venous phenotype [48]. While ECFCs and mature endothelial cells share a similar phenotype, arterial–venous–lymphatic specifications of ECFCs remain controversial. Nonetheless, subtle transcriptome differences between ECFCs and mature endothelial cells significantly impact on the behavior of the cells. In vitro experiments have suggested that ECFCs may have stronger angiogenic potential compared to mature endothelial cells [49, 50].

### Comparing the characteristics of ECFCs from different sources

ECFCs from different sources maintain some distinct features, which is supported by several studies. DiMaio et al. performed high-throughput sequencing to identify the DEGs in blood and lymphatic ECFCs. For example, neuregulin 1 was the most upregulated gene in blood ECFCs compared to lymphatic ECFCs. Pathway analysis of the top DEGs in the two groups revealed that these DEGs played an important role in regulation of cell differentiation, vasculature development, and endothelial cell differentiation [46]. In vitro studies demonstrated superior angiogenic capacity in CB-ECFCs compared to PB-ECFC [22, 51]. However, the regulatory factors affecting angiogenesis are not fully understood. Khoo et al. conducted miRNA sequencing to evaluate the transcriptional difference of CB-ECFCs and PB-ECFCs to determine the regulatory factors affecting angiogenesis. A total of 50 miRNAs were identified as differently expressed miRNAs. Among them, miR-193a-3p was more highly expressed in PB_ECFCs than CB_ECFCs. miR-193a-3p could inhibit the migration and angiogenesis ability of CB-ECFCs by targeting HMGB1 [52]. Additionally, Liu et al. analysed the integrated datasets of CB-ECFCs and PB-ECFCs to investigate their osteogenic and angiogenic potential. CB-ECFCs had higher expression of osteogenic and angiogenic genes, including BMP-1, BMP-2, BMP-4, BMP-6, TGF-β2, and COL1A1 [53]. In addition, the characteristics of ECFCs isolated from human term PL have been explored by comparing with CB-ECFCs. There were only 33 DEGs between PL-ECFCs and CB-ECFCs. Although PL-ECFCs and CB-ECFCs did not show differences in proliferation and angiogenesis capacity, some angiogenesis related genes (MFGE8, FOXC2, SMAD6, and IGFBP2) were highly enriched in CB-ECFCs [17]. The exploration of ECFCs from different tissues will help to understand their different functional abilities and discover angiogenesis-related genes.

### Extracellular vesicles of ECFCs

**Fig. 4:**
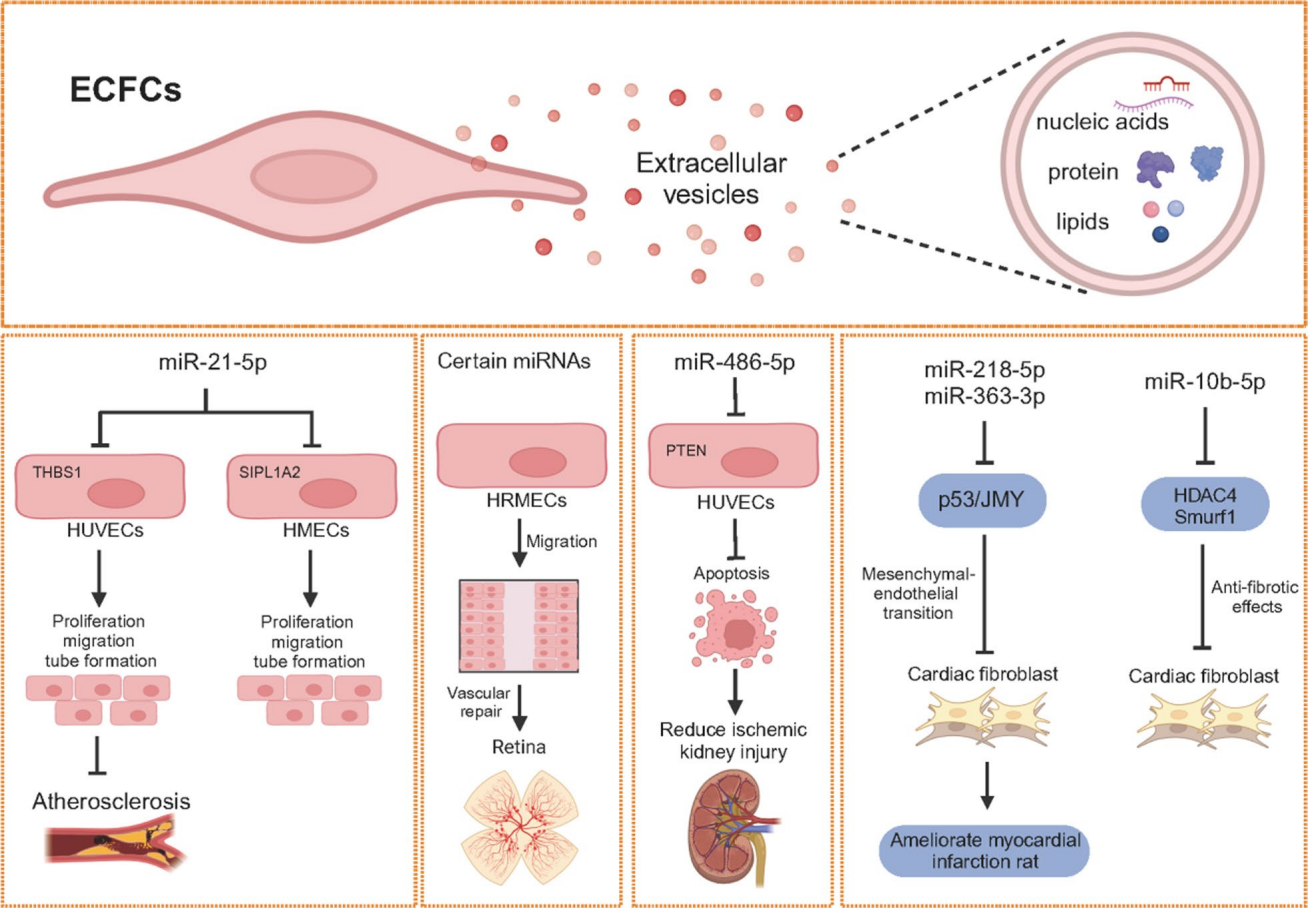
ECFCs-secreted extracellular vehicles (EVs) therapy for various diseases. As shown in the figure above, ECFC EVs act on target cells show a positive effect on these diseases

Extracellular vesicles (EVs) are membrane-contained vesicles secreted by most cell types [54] that can transfer bioactive molecules to mediate intercellular communication, including lipids, nucleic acids (mRNAs and miRNAs) and proteins [55]. These substances facilitate intercellular signalling by modulating gene activity and cellular function in target cells that take up EVs. Several research groups have sequenced ECFC EVs to investigate their roles and molecular mechanisms in various biological processes (Fig. 4). For instance, Deregibus et al. characterised the specific miRNA signature of CB-ECFC EVs when compared with CB-ECFCs. In vivo and in vitro experiments demonstrated that CB-ECFC EVs contributed to vascular repair. They found that miR-21-5p was significantly higher in CB-ECFC EVs compared to that of CB-ECFCs, which promoted proliferation, migration, and angiogenesis of endothelial cells by blocking THBS (a negative regulator of angiogenesis) [56]. Similarly, Ke et al. compared the miRNA expression between PB-ECFCs EVs and the parental PB-ECFCs. They found 100 upregulated miRNAs and 285 downregulated miRNAs between PB-ECFCs and PB-ECFCs EVs. The reverse transcription-polymerase chain reaction (RT-PCR) showed that miR-122-5p, miR-451a, miR-6087, miR-363-3p, miR-486-5p, and miR-218-5p were upregulated while miR-181-3p, miR-500a-3p, miR-362-5p, miR-21-3p, miR-374a-3p, and miR-365a-5p were downregulated in PB-ECFCs EVs. Notably, exosomal miR-218-5p and miR-363-3p promoted the proliferation, angiogenesis, and mesenchymal-endothelial transition of cardiac fibroblasts. Additionally, exosomal miR-218-5p and miR-363-3p were able to ameliorate mice with myocardial infarction by targeting the p53/JMY signalling pathway [57].

Another study described the differential miRNA expression pattern of CB-ECFC EVs and CB-ECFC microparticles. miR-486-5p was identified as the most abundant miRNAs in ECFC EVs. CB-ECFC EVs prevented apoptosis of endothelial cells under hypoxia by increasing miR-486-5p, decreasing PTEN, and stimulating Akt phosphorylation. The transfer of ECFC-EVs with enriched miR-486-5p to the kidney demonstrated protective effects against ischemic kidney injury [58]. Liu et al. performed miRNA profiling analysis of PB-ECFC EVs, which showed 1861 differentially expressed miRNAs in EVs generated from PB-ECFCs under normal oxygen (nor-exo) and hypoxia (hyp-exo). The nor-exo EVs were enriched with miR-10b-5p, which had anti-fibrotic effects on cardiac fibroblasts by targeting the fibrotic genes Smurf1 and HDAC4 [59].

Additionally, based on the miRNA microarray analysis, Xiao et al. reported that miR-21-5p was one of the most abundant miRNAs in PB-ECFC EVs. miR-21-5p in the PB-ECFC EVs decreased SIPA1L2 expression to increase autophagy and showed the protective effects in HMECs damage caused by oxidized low density lipoprotein. More importantly, PB-ECFC EVs with miR-21-5p alleviated vascular injury, lipid metabolism disorders, and activated autophagy in the atherogenic rat model [60]. In the oxygen-induced retinopathy model, PB-ECFC EVs contributed to the vascular repair in the retina. In vitro, CB-ECFC EVs enhanced the migration of human retinal microvascular endothelial cells (HRMECs) and altered the expression level of genes that were involved in pathways associated with ‘development of vasculature’ and ‘angiogenesis’. Analysis of small RNA sequencing data revealed top 5 miRNAs enriched in ECFC EVs compared to ECFCs, namely miR-4532-5p, miR-451a-5p, miR-7704-5p, miR-486-2-5p, and miR-486–1-5p. Conversely, the top five miRNAs overrepresented in the CB-ECFCs were miR-374a-3p, miR-100-3p, miR-27a-5p, let-7e-5p, and let-7d-5p. Furthermore, the target genes of the top 15 enriched miRNAs were involved in ‘metastasis’, ‘development of vasculature’, ‘angiogenesis’ and ‘cell movement of endothelial cells’ [61].

Nevertheless, the mechanism behind the therapeutic effect of ECFC EVs is still not fully known. Besides the most abundant miRNA in ECFC EVs, other miRNAs or proteins may also contribute to the therapeutic effects of ECFC EVs. Engineered EVs with increased miRNA levels should be considered to achieve a better therapeutic effect [62].

### Understanding ECFC changes in disease and investigating disease specific cell modification for therapeutic translation

Although therapeutic interventions using ECFCs have been shown to be beneficial in preclinical settings, ECFCs cytotherapy has yet to be successfully introduced in the clinical arena. Given the complex pathophysiology of ECFCs in some diseases, large scale approaches to identification of multiple signalling networks in dysfunctional ECFCs might be more successful in the search for novel diagnostic or therapeutic targets. In the following section, we will focus on the variations in gene expression profiles of ECFCs under various kinds of pathologic states.

#### Hypoxia

To date, much of the research investigating the efficacy of ECFCs for therapeutic use has focused on ischaemic conditions such as critical limb ischaemia, CAD, and stroke. Considering that ECFCs would be exposed to hypoxia when transplanted into ischemic tissues, it is necessary to investigate the alternations of ECFCs under hypoxia. Tasev et al. found that hypoxia inhibited the initial outgrowth of ECFCs and the proliferation of cultured PB-ECFCs. They then mapped the transcriptional signatures of PB-ECFCs cultured under 1% O_2_ to explore the potential mechanisms. Gene set enrichment analysis of upregulated genes revealed that the most significantly altered pathways were related to amino acid metabolism, glycolysis/gluconeogenesis, and carbon-, fructose- and mannose metabolism. The downregulated genes were involved in cell cycle, p53 signalling pathway and cytokine-cytokine receptor interactions [63]. Another study examined the gene expression profiles of CB-ECFCs under hypoxic conditions (5% O_2_) for 3, 6, 12, 24, and 48 h duration. The results revealed that DEGs of CB-ECFCs exposed to hypoxia were involved in cell apoptosis, cell cycle and mitogen-activated protein kinase pathways [64].

Similarly, the cell transcriptional responses of CB-ECFCs exposed to hypoxia (1% O_2_) for 48 h were examined using microarray. As expected, HIF-1α signalling and glycolysis pathway were activated in CB-ECFCs. The most significant upregulated genes were ANKRD37, AK3L1, PLAC8, and ANGPTL14, and the most significant downregulated included ELMOD1 and LYVE1 in hypoxia-treated CB-ECFCs. More importantly, the study demonstrated that silencing of PLAC8 or overexpression of NOX4 (a downstream target of PLAC8) improved CB-ECFC angiogenic ability when exposed to hypoxia [65]. Guduric et al. performed bulk RNA sequence analysis of CB-ECFCs after 24 h exposure to 1% O_2_. ANGPTL14, ENO2, ETXNIP, and SLC2A3 were upregulated while VEGFR2, NOS3, and FLT1 were downregulated. Although the HIF1 pathway was activated, there was no significant enrichment for VEGFA pathway [66]. These studies suggest that a hypoxic microenvironment (1–5% O_2_) can activate HIF1, glycolysis, and cell apoptosis pathways, impairing the function of ECFCs. It may be essential to use genetic manipulation or bioactive compounds to enhance ECFC function in the setting of hypoxia.

#### Premature neonates and pregnancy-related disease

Several studies have demonstrated that CB-ECFCs isolated from low birth weight (LBW) and preterm (PT) infants exhibited reduced angiogenesis capacity or accelerated senescence [67–69]. Simoncini et al. conducted a microarray analysis of ECFCs from 4 term born and 11 PT infants. MKK6-p38MAPK pathway was predicted to be activated in PT-ECFCs. Further experiments revealed that SIRT1 deficiency controlled the activation of MKK6-p38MAPK pathway by increasing MKK6 expression, thereby leading to the senescence-associated secretory phenotype of PT-ECFCs. Treatment with resveratrol (a SIRT1 activator) and SB203580 (a p38MAPK activity inhibitor) could reverse the senescence-associated secretory phenotype of PT-ECFCs [67].

CB-ECFCs function may also be affected by maternal features and pregnancy related disease. Dysfunctional CB-ECFCs have been observed in mothers with high gestational weight gain (GWG) [70], preeclampsia [71, 72] and gestational diabetes mellitus (GDM) [73, 74]. CB-ECFCs isolated from pregnancies with a GWG of > 13 kg demonstrated reduced wound healing ability compared to those isolated from pregnancies with a GWG of < 13 kg. RNA sequencing identified the killer cell lectin-like receptor K1 antisense RNA (KLRK1-AS1) as a key factor linked to CB-ECFCs wound healing function. Low KLRK1-AS1 expression level would lead to the impaired wound healing of CB-ECFCs [70].

CB-ECFCs from preeclampsia pregnancies had a different genomic methylation pattern compared to those from normal pregnancies. A total of 1266 CpG sites in passage 3 and 2362 sites in passage 5 were found in CB-ECFCs from preeclamptic pregnancies. Differentially methylated genes may play a role in cell metabolism, cell cycle and transcription, cell–cell interaction and Wnt signalling [75]. Additionally, investigations on the miRNA profiles of CB-ECFCs from six preeclamptic pregnancies and six healthy pregnancies revealed a series of differentially expressed miRNAs, such as miR-2467-5p and miRNA-1270 [76]. Downregulation of miRNA-1270 can lead to increased level of ataxia telangiectasia mutated(ATM) and VE-cadherin phosphorylation, thereby impairing the CB-ECFCs function. Increasing miR-1270 and/or downregulating ATM may serve as potential therapeutic targets in preeclamptic CB-ECFCs [77]. A study explored the aberrantly expressed genes of CB-ECFCs from GDM and reported an upregulation of PLAC8. Bisulfite sequencing was performed to explore the epigenetic alterations in GDM-exposed CB-ECFCs. The results showed that DNA hypomethylation in intron 1 [5] of PLAC8 was involved in the upregulated PLAC8 expression of GDM-exposed CB-ECFCs. Silencing PLAC8 was shown to improve proliferation and senescence of GDM-exposed CB-ECFCs [78].

These studies highlight the altered genes of dysfunctional ECFCs in PT infants, and pregnancies with GWG, preeclampsia, and GDM. Further studies are required to determine the relationship between dysfunctional ECFCs and increased cardiovascular risk which may be associated with these diseases.

#### Coronary artery disease

ECFC dysfunction is correlated with the onset of cardiovascular disorders, especially CAD [79–81]. The miRNA sequencing analysis showed a significant upregulation of miR-410-3p, miR-497-5p, and miR-2355-5p in CAD-ECFCs. The three miRNAs acted as negative regulators of angiogenesis by silencing expression of VEGFR2. Knockdown of these three miRNAs improved the tube formation ability of CAD-ECFCs in vitro and accelerated limb blood flow recovery in a mouse hindlimb ischemia model [82]. The miRNA expression profile of ECFCs from control and patients with CAD was performed to identify miRNA candidates that were dysregulated in CAD-ECFCs. Two miRNAs have been found to be upregulated in both plasma and ECFCs from patients with CAD: miR-146a-5p and miR-146b-5p, which both targeted an angiogenesis-related gene RHOJ. Furthermore, knockdown of miR-146a-5p and miR-146b-5p could improve the migration and tube formation activity of CAD-ECFCs. By contrast, overexpression of miR-146a-5p and miR-146b-5p inhibited migration and tube formation activity of ECFCs from control group [83]. Even though knockdown of these miRNAs have shown positive findings in CAD-ECFCs, further research is needed to develop vehicles for targeted delivery of miRNAs to ECFCs with few adverse effects.

#### Diabetes mellitus (DM)

It has been reported that the function of ECFCs are impaired in patients with diabetes, including proliferation, migration, and tube formation [84, 85]. Shen et al. performed microarray analysis of PB-ECFCs from five healthy donors and nine diabetic patients. A total of 822 upregulated and 148 downregulated genes were identified as DEGs, which were mainly accumulated in inflammation‑associated pathways. Among these DEGs, IL8 and CXCL1 were significantly upregulated in DM-ECFCs [86]. Similarly, Tan et al. compared the function of PB-ECFCs from healthy donors and patients with proliferative diabetic retinopathy (PDR). They found that PDR-ECFCs showed impaired ability to migrate towards stromal cell-derived factor-1 (SDF-1) and inefficient incorporation with human retinal endothelial cells. The microarray analysis of healthy-ECFCs and PDR-ECFCs revealed two anti-angiogenic genes, TSP-1 and TIMP-3 were upregulated in PDR-ECFCs [87]. Collectively, the increased inflammatory response and anti-angiogenic factors may contribute to the dysfunctional ECFCs in DM.

#### Pulmonary arterial hypertension (PAH)

Researchers have found that PB-ECFCs from patients with PAH are hyperproliferative and correlate with markers of disease severity [88]. miRNAs provided some insight into the unclear mechanisms underlying the proliferative angiopathic process in PAH. Paola et al. analyzed the miRNA profile of two types of pulmonary arterial hypertension (PAH): heritable PAH (HPAH) and idiopathic PAH (IPAH). HPAH is caused by mutations in the bone morphogenetic protein receptor type 2 (BMPR2) gene while the cause of IPAH is still unclear. miR-124 was identified as the most significantly downregulated gene in both IPAH and HPAH when compared to healthy donors. The reduced expression of miR-124 was also observed in a rat model of PAH. In vitro experiments demonstrated that transfection of miR-124 mimics partially restored mitochondrial activity of PB-ECFCs from PAH patients and reduced the expression of glycolysis related genes (MCT1, LDHA, PDK1, and PDK2) and proliferative abnormalities of these cells [89]. A study has reported that miR-124 was able to inhibit the proliferation of pulmonary vascular smooth muscle cells and the development of PAH by targeting NFATc1, CAMTA1, and PTBP1 [90]. Thus, the overexpression of miR-124 might be a promising therapeutic approach for the treatment of PAH.

#### Cancers

ECFCs were capable of forming capillary structures in vitro and in vivo and showed an innate tumour tropism [91]. In addition, ECFC invasion and tubular formation may attenuate the effects of anti-angiogenic therapies to tumours [92]. Moccia and colleagues performed the gene analysis of PB-ECFCs from healthy donors and breast cancer (BC), and renal cell carcinoma (RCC). In BC-ECFCs, SULF1, CXCL10 and FCGR2A were significantly upregulated while CBC1 and PTPN22 were significantly downregulated. In RCC-ECFCs, SULF1, IFGBP1 and FOS were identified as upregulated genes while PLXCD3, NTSR1 and PTPN22 were identified as downregulated genes. More importantly, BC-ECFCs and RCC-ECFCs shared 35 DEGs. FOS was predicted to be the hub gene of the 35 shared DEGs [93]. The shared gene signature could serve as candidate genes for the anti-angiogenic treatment in BC and RCC.

#### Myelodysplastic syndromes (MDS)

MDS is a kind of myeloid neoplasm caused by clonal mutation of haematopoietic stem cells, which leads to abnormal proliferation, failure of haematopoiesis in the bone marrow and may progress to acute myeloid leukemia [94]. It has been reported that there was significantly higher number of ECFCs colonies in patients with MDS compared to healthy controls. Besides, ECFCs from MDS patients exhibited increased adhesion capacity compared to that from healthy controls. According to the gene expression profiles, several genes related with cell adhesion were upregulated in MDS ECFCs while several genes of the Wingless and int (Wnt) pathways were downregulated. Additionally, certain miRNAs regulating the Wnt pathway were decreased in MDS ECFCs. Supplementation of the Wnt ligand Wnt3A both reduced expression of genes related with cell adhesion and partially restored cell differentiation [95]. Taken together, the defective expression level of Wnt pathway components may lead to the dysfunction of ECFCs in MDS and the addition of Wnt3A was an effective approach for restoring the function of MDS ECFCs.

#### Haematological system diseases and vascular diseases

##### Von willebrand disease (VWD)

VWD is the most common inherited bleeding disorder and is caused by quantitative (types 1 and 3) or qualitative (type 2) defects of VWF [96]. ECFCs have been utilized to investigate the pathophysiology of VWD. In a study, transcriptome-wide differences of ECFCs between control donors and patients with type 1 VWD revealed 64 mRNAs and 7 miRNAs that were differentially expressed in ECFCs between control donors and type 1 VWD patients during basal VWF release. On the other hand, during stimulated VWF release, 190 mRNAs and 5 mRNAs were differentially expressed in ECFCs from control donors and type 1 VWD patients respectively [97]. Using single cell sequence, the Ng lab identified the DEGs between ECFCs isolated from donors with low VWF levels and control endothelial cells. FLI1 was identified as the candidate gene that mediated the expression level of VWF, therefore future work can investigate modulating FLI1 expression to restore VWF in type 1VWD [48].

##### Venous thromboembolism (VTE)

VTE is the third most common cause of vascular mortality worldwide [98]. 25% to 50% of VTE events are classified as unprovoked VTE (uVTE) due to the lack of predisposing conditions. ECFCs have been utilized to explore the pathogenic mechanisms of uVTE. Microarray gene expression analysis identified 2905 DEGs between control ECFCs and uVTE ECFCs. Among DEGs, the anti-angiogenic cytokine TNFSF15 and its death-receptor TNFRSF25 were upregulated in uVTE ECFCs. Upregulation of TNFSF15–TNFRSF25 axis impaired survival and proliferation of uVTE ECFCs. TNFSF15 blocking antibody significantly increased the proliferation and angiogenic ability of uVTE ECFCs [99]. In summary, TNFSF15–TNFRSF25 axis may represent a novel target for the treatment of uVTE patients.

##### Moyamoya disease (MMD)

MMD is a cerebrovascular disease, characterized by idiopathic and progressive occlusion of the major bilateral intracranial arteries with the compensatory formation of capillary collaterals [100]. Dysfunctional ECFCs may be involved in the pathogenesis of MMD. A group found that tube formation ability was impaired in MMD PB-ECFCs and they identified 537 DEGs between PB-ECFCs from MMD and normal controls. The most downregulated gene in MMD PB-ECFCs, RALDH2, was selected for subsequent experiments. The downregulation of RALDH2 was caused by defective acetyl-histone H3 (Ac-H3) binding to the promoter region [101]. Notably, further experiments revealed that a histone deacetylase (HDAC) inhibitor, panobinostat, effectively upregulated Ac-H3 and RALDH2 expression, thereby restoring the angiogenic potential of MMD PB-ECFCs [102]. In addition, the same lab found that CDKN2A may also contribute to the dysfunctional PB-ECFCs in MMD [103]. However, although knockdown of CDKN2A restored the cell growth and tubule formation ability of MMD PB-ECFCs, it may increase the risk for tumor formation. Except the altered gene expression profiles in MMD PB-ECFCs, epigenetic regulation has been reported to mediate the function of MMD PB-ECFCs. Sung et al. investigated the DNA methylation profiles of PB-ECFCs from control and MMD group. Hypomethylation at the SORT1 promoter CpG sites was observed in PB-ECFCs from MMD group, leading to the increased expression level of SORT1. DNA methylation status at the SORT1 promoter CpG site in PB-ECFCs could be used to distinguish patients with MMD from control donors. SORT1 overexpression was found to inhibit the tube formation of HUVECs [104]. These results suggested that aberrant promoter hypomethylation of SORT1 may contribute to the pathogenesis of MMD.

## Conclusions and future perspectives

Transcriptome-sequencing tools have contributed significant advances to the ECFC research, including revealing the origin of ECFCs, characterising the phenotype and functional characteristics of ECFCs, and discovering the key molecules and pathways of ECFCs under disease states. Single cell sequence and pseudotime analysis suggested that ECFCs may originate from blood vessel walls. Transcription analysis provides evidence that ECFCs are different from mature endothelial cells and they have distinctive characteristics when isolated from different tissues. The gene expression pattern of ECFC-derived EVs can elucidate the molecular pathways underlying their therapeutic potential. Moreover, transcriptional signatures of ECFCs in different diseases undoubtedly contribute to the precision and convenience of drug target discovery. However, there are three major issues that need attention. First, it is essential to emphasize that the key molecules and pathways identified in transcriptomics in various diseases necessitate additional validation by external datasets and rigorous functional assays. Second, the integrative analysis of multiple datasets needs algorithms to remove the batch effects to obtain reliable results [105–107]. Third, the heterogeneity and dynamics of ECFCs during developmental processes and the progression of disease still require further investigation. It is of interest to explore the following aspects using high throughput technology: (i) establish the standard marker gene panel of ECFCs; (ii) obtain distinguishing markers of ECFCs with high proliferative potential [3]; (iii) identify different ECFCs subtypes and their functions; (iv) identify the key regulatory factors of ECFCs subtypes with high proliferative ability and enhance therapeutic effects of ECFCs; (vi) reveal ECFCs crosstalk with other cell types using single cell and spatial transcriptomics.

Although transcriptome profiling has remodelled the understanding of ECFCs, multi-omics approaches combining transcriptomics, proteomic [108–111] and metabolomic mass spectra will help to better understand how genes, proteins, metabolic activities, and their interaction influence the functionality of ECFCs. CRISPR-Cas9 for gene editing in ECFCs and bio-printing of vascular structures may improve the angiogenic ability of ECFCs exposed to the ischemic and hypoxic environment. ECFC-derived EVs have inherent advantages because they will not be affected by ischemic and hypoxic environments. Integration of multi-omics data, application of ECFC-derived EVs, CRISPR-Cas9 for gene editing in ECFCs [112], and bio-printing of vascular structures [113] represent major frontiers for future exploration. A comprehensive understanding of ECFC biology and combination of emerging technologies may contribute to the successful clinical translation of ECFCs in the furfure.

## Data Availability

Not applicable.

## Abbreviations

Ac-H3: Acetyl-histone H3
AT-EC: Endothelial cell isolated from adipose tissue
ATM: Ataxia telangiectasia mutated
BC: Breast cancer
CAD: Coronary artery disease
CBMNCs: Cord blood mononuclear cells
CFU-ECs: Endothelial cell colony-forming units
DEGs: Differently expressed genes
DM: Diabetes mellitus
ECFCs: Endothelial colony forming cells
EPCs: Endothelial progenitor cells
EVs: Extracellular vesicles
GDM: Gestational diabetes mellitus
GO: Gene ontology
GWG: Gestational weight gain
HCAECs: Human coronary artery endothelial cells
HRMECs: Human retinal microvascular endothelial cells
HMECs: Human microvascular endothelial cells
HUVECs: Human umbilical vein endothelial cells
JAK2: Janus kinase 2
KLRK1-AS1: Killer cell lectin-like receptor K1 antisense RNA
LBW: Low birth weight
MACs: Myeloid angiogenic cells
MDS: Myelodysplastic syndromes
MMD: Moyamoya disease
PAH: Pulmonary arterial hypertension
PBMNCs: Peripheral blood mononuclear cells
PDR: Proliferative diabetic retinopathy
PL: Placenta
PT: Preterm
RT-PCR: Reverse transcription-polymerase chain reaction
PROCR: Protein C receptor
RCC: Renal cell carcinoma
uVTE: Unprovoked venous thromboembolism
VTE: Venous thromboembolism
VWD: Von Willebrand disease
vWf: Von Willebrand factor
Wnt: Wingless and int
